# Cæsarean Section

**Published:** 1891-03

**Authors:** Seth M. Morris

**Affiliations:** Student, N. Y.; Austin


For Daniel's Texas Medical Journal.

### CÆSAREAN SECTION

### At Roosevelt Hospital, N. Y., by Dr. G. M. Tuttle.

REPORTED BY SETH M. MORRIS, STUDENT, N. Y.

THE WOMAN had been in labor about twenty hours. On examination at the time of the operation, the child was found to be dead, the cord prolapsed and a hand and foot presenting from the cervix. Its head was in the left iliac fossa well above the brim, the breech to the right side and the uterus tonically contracted. Unsuccessful efforts had been made by a skillful obstetrician to turn the child, and further efforts were desisted from for fear of rupturing the uterus. The pelvic measurements gave

27¼ cm. for the interspinous which is 1¼ cm. above the average, 31 cm between the crests, and for the conjugata vera about 6 cm. It was decided to do Cæsarean section. The strictest antiseptic precautions were observed. The vagina was douched out thoroughly and the abdominal wall cleansed with soap and water, alcohol and bichloride solution. An incision was then made about 8 inches in length in the median line over the most prominent part of the uterus. The peritoneum was incised for the same distance and sponges stuffed between the uterine and abdominal walls in order to protect the peritoneal cavity. A six inch incision was made in the uterus and the child rapidly extracted together with the placenta and membranes. An assistant in the meantime controlled the hemorrhage by grasping the broad ligaments with his hands and compressing them. The cavity of the uterus, now very much contracted since the removal of the child, was packed with sponges and douched with hot water. The operator began to stitch up the uterus with interrupted sutures of large silk passed through the muscular and peritoneal coats only, but the uterus could not be made to contract sufficiently to stop the hemorrhage, and it was decided to remove it. A large elastic ligature was passed around its lower segment and tied tightly, which stopped the hemorrhage. The uterus was then amputated a little above the ligature, and the stump transfixed by two large needles. The peritoneum at the lower end of the incision was stitched closely around the stump shutting off the abdominal cavity, and the remainder of the incision in it stitched up by interrupted cat gut sutures. The wound in the abdominal wall was stitched up by a continuous suture of large silk supplemented by interrupted sutures of silk worm gut. The uterine stump, transfixed by the two large needles which prevented it from receding into the abdomen, was fixed in the lower part of the abdominal wound. A good sized glass drainage tube, reaching well to the bottom of the pelvis, was put in about two inches above the uterine stump. The stump was cauterized with the Paquelin cautery, and it and the abdominal wound dressed with iodoform gauze, sublimate gauze, cotton and a wide abdominal bandage. The ovaries and tubes were also removed with the uterus.

